# Research Progress of Computational Fluid Dynamics in Mixed Ionic–Electronic Conducting Oxygen-Permeable Membranes

**DOI:** 10.3390/membranes15070193

**Published:** 2025-06-27

**Authors:** Jun Liu, Jing Zhao, Yulu Liu, Yongfan Zhu, Wanglin Zhou, Zhenbin Gu, Guangru Zhang, Zhengkun Liu

**Affiliations:** 1 State Key Laboratory of Materials-Oriented Chemical Engineering, College of Chemical Engineering, Nanjing Tech University, 30 Puzhu Road(S), Nanjing 211816, China; liujun-7@njtech.edu.cn (J.L.); zj2023@njtech.edu.cn (J.Z.); yululiu9622@163.com (Y.L.); 202262104053@njtech.edu.cn (Y.Z.); wlzhou@njtech.edu.cn (W.Z.); guzhenbin@njtech.edu.cn (Z.G.); guangru.zhang@njtech.edu.cn (G.Z.); 2 Quzhou Membrane Material Innovation Institute, Nanjing Tech University, Quzhou 324000, China

**Keywords:** mixed-conducting membrane, CFD, oxygen separation, membrane reactors, membrane modules

## Abstract

Mixed ionic–electronic conducting (MIEC) oxygen-permeable membranes have emerged as a frontier in oxygen separation technology due to their high efficiency, low energy consumption, and broad application potential. In recent years, computational fluid dynamics (CFD) has become a pivotal tool in advancing MIEC membrane technology, offering precise insights into the intricate mechanisms of oxygen permeation, heat transfer, and mass transfer through numerical simulations of coupled multiphysics phenomena. In this review, we comprehensively explore the application of CFD in MIEC membrane research, heat and mass transfer analysis, reactor design optimization, and the enhancement of membrane module performance. Additionally, we delve into how CFD, through multiscale modeling and parameter optimization, improves separation efficiency and facilitates practical engineering applications. We also highlight the challenges in current CFD research, such as high computational costs, parameter uncertainties, and model complexities, while discussing the potential of emerging technologies, such as machine learning, to enhance CFD modeling capabilities. This study underscores CFD’s critical role in bridging the fundamental research and industrial applications of MIEC membranes, providing theoretical guidance and practical insights for innovation in clean energy and sustainable technologies.

## 1. Introduction

Mixed ionic–electronic conducting (MIEC) oxygen-permeable membranes have garnered significant attention in fields such as oxygen separation, energy conversion, and environmental protection [1,2,3,4,5]. These membrane materials exhibit unique dual conductivity, enabling selective oxygen ion and electron conduction at high temperatures, thereby facilitating oxygen permeation without needing an external power source. This property positions MIEC membranes as crucial materials for oxygen separation processes, especially in applications such as oxy-fuel combustion [6,7,8,9], partial oxidation reactions [10,11,12], and hydrogen production [13,14,15,16]. Compared to traditional oxygen separation methods, MIEC membranes can substantially reduce energy consumption, providing a more environmentally sustainable solution. Consequently, optimizing the performance, stability, and durability of MIEC membranes in practical applications has become a central focus in current research, particularly as the global industry shifts toward efficient and green technologies.

Computational fluid dynamics (CFD) is a powerful numerical tool widely used in the membrane field [17,18,19,20,21]. It is possible to optimize and improve the performance of MIEC membranes by using CFD to simulate complex transport processes, including multiphysical phenomena such as heat transfer, mass transfer, and chemical reactions. These processes typically occur within membranes in high-temperature, reactive atmospheres and within those with complex geometries, making them exceedingly challenging or impossible to observe experimentally. CFD provides valuable multidimensional data on the oxygen partial pressure, temperature distribution, velocity fields, and other characteristics within MIEC membrane systems through numerical modeling and simulation. These insights are essential for optimizing membrane materials and developing new membrane structures to enhance oxygen permeation efficiency and operational performance.

Specifically, CFD helps researchers understand the micro- and macroscopic oxygen transport mechanisms within MIEC membranes. CFD simulations offer detailed analyses of factors affecting oxygen permeation rates, including operational temperature, material properties, oxygen partial pressure gradients, and the gas composition in the reaction zone. In MIEC membranes, temperature and gas concentration mainly influence oxygen ion and electron migration. CFD quantifies the impacts of these factors, providing theoretical support for material selection and structural optimization. Furthermore, CFD can be used to evaluate membrane performance in specific application environments and to propose strategies to minimize material degradation and enhance membrane longevity by identifying hotspot regions or areas of localized stress concentration.

Numerous studies have employed CFD to analyze the role of MIEC membranes in oxygen separation and fuel cell applications [22,23,24]. Early CFD models were relatively simple, primarily focusing on single-phase flow and diffusion mechanisms, often neglecting the complexities of reactive coupling processes. However, recent computational capabilities and CFD software advancements have enabled the development of more sophisticated multiphysics models, where chemical reactions, heat transfer, and mass transfer processes can be examined simultaneously. Such models can more accurately represent the actual working conditions of the membranes, allowing researchers to better predict and understand their behavior in complex environments. For instance, through multiscale modeling, CFD can reveal the transport behavior of ions and electrons within the membrane material at the microscopic level while examining the influence of gas distribution and flow patterns on membrane performance at the macroscopic level.

**Figure 1:**
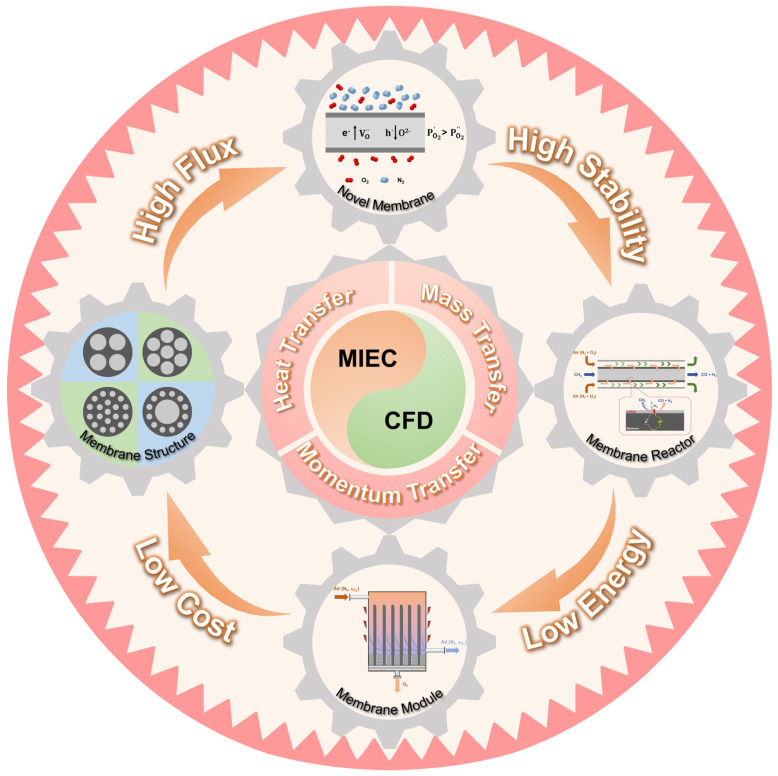
The main application of CFD in MIEC oxygen-permeable membranes.

In this review, we aim to provide a comprehensive overview of CFD applications in MIEC oxygen-permeable membrane research, covering foundational studies and the latest advancements in the field (Figure 1). We focus on various CFD modeling approaches and their applications in membrane processes. We discuss the mathematical frameworks, unique characteristics of CFD models for membrane processes, and the impact of different flow patterns (e.g., cross-flow, counter-flow, and co-flow) on membrane performance. By examining these aspects, we demonstrate CFD’s contributions to optimizing MIEC membrane design and functionality, with a particular emphasis on (i) oxygen permeation mechanisms, (ii) heat and mass transfer, and (iii) membrane reactor and module design. Additionally, we address the challenges in CFD modeling, such as high computational costs, limitations in model accuracy, and scaling issues, and we propose potential directions for further enhancing CFD applications in this field.

## 2. Fundamentals of MIEC Oxygen-Permeable Membranes

MIEC membranes are advanced materials that have garnered significant interest for their potential in high-efficiency oxygen separation applications. These membranes uniquely combine ionic and electronic conductivity, enabling selective oxygen permeation at elevated temperatures, typically above 600 °C [25,26]. The oxygen permeation process in MIEC membranes is driven by a partial pressure gradient and is supported by the inherent mixed conductivity, making them valuable for various applications, including oxy-fuel combustion, syngas production, and industrial oxygen supply.

**Figure 2:**
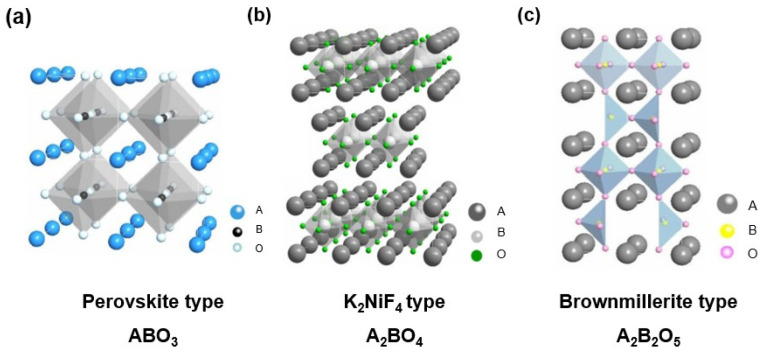
Crystal structures of typical MIEC oxygen-permeable membrane materials: (**a**) perovskite type, (**b**) K_2_NiF_4_ type, and (**c**) brownmillerite type. Reprinted with permission from Ref. [29]. Copyright 2013 China Science Press.

MIEC membranes typically comprise ceramic materials, particularly perovskite, perovskite-like, and brownmillerite-type oxides. The perovskite structure (Figure 2a), characterized by an ABO_3_ configuration, where A and B are metal ions, exhibits excellent structural flexibility. This adaptability facilitates oxygen ion migration through the formation of oxygen vacancies in the lattice, critical for enabling oxygen permeation. Moreover, perovskite-structured materials demonstrate outstanding thermal and chemical stability in high-temperature and reactive atmospheres, making them up-and-coming candidates for MIEC oxygen-permeable membranes. Notable examples include Ba_0.5_Sr_0.5_Co_0.8_Fe_0.2_O_3−δ_ (BSCF) [27] and La_0.6_Sr_0.4_Co_0.8_Fe_0.2_O_3−δ_ (LSCF) [28]. Perovskite-like structures (Figure 2b), such as those with a K_2_NiF_4_ configuration (A_2_BO_4_ materials), are exemplified by La_2_NiO_4_. In these materials, oxygen transport occurs through interstitial positions. Brownmillerite-type oxides (Figure 2c), characterized by an A_2_B_2_O_5_ structure, are mixed conductors with ordered oxygen vacancies. While these materials offer relatively high structural stability, their oxygen permeation performance is comparatively low.

**Figure 3:**
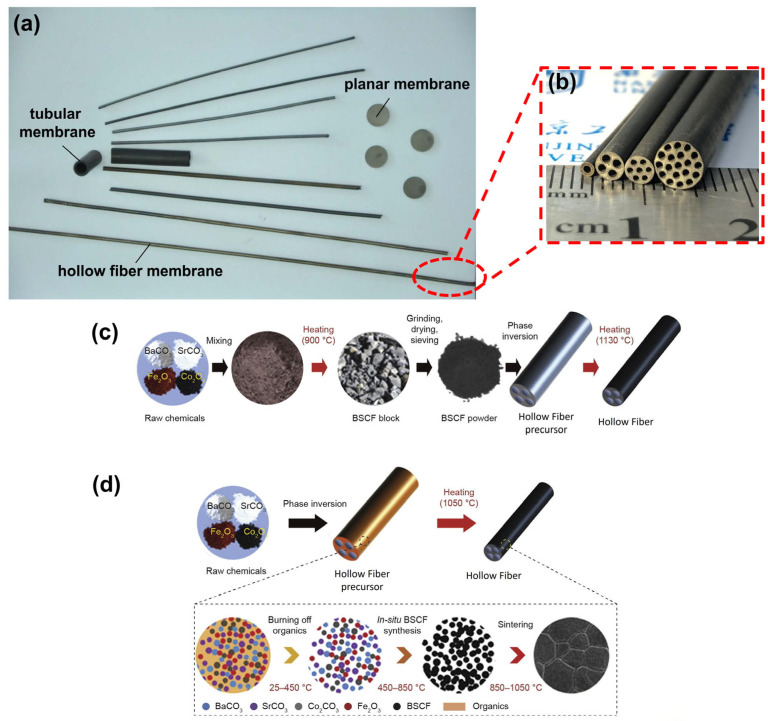
Images of (**a**) planar, tubular, and hollow fiber membranes, reprinted with permission from Ref. [30], copyright 2013 China Science Press. (**b**) A single channel, four channels, seven channels, and nineteen channels. A schematic of perovskite hollow fiber membrane fabrication approaches: (**c**) conventional multistep approach and (**d**) one-step thermal processing (OSTP) approach, reprinted with permission from Ref. [34], copyright 2017 John Wiley and Sons.

Based on specific applications, the operating environment, and the required oxygen flux, MIEC membranes can be designed in various structural forms, including planar, tubular, and hollow fiber forms (Figure 3a). In industrial applications, planar membranes face challenges such as limited effective oxygen-permeable surface areas, difficulties in module connection, and unresolved issues with high-temperature sealing [30]. With their longer lengths, tubular membranes can utilize cold-end sealing to address high-temperature sealing issues. However, their relatively thick walls result in a low packing density and a higher oxygen permeation resistance, which limits their scalability for large-scale applications [31]. In recent years, hollow fiber oxygen-permeable membranes have gained attention due to their unique asymmetric structure, comprising a porous support layer and a thin, dense layer [32,33]. This design offers a high packing density and low permeation resistance, making hollow fiber membranes highly promising for future engineering applications. However, the thin membrane layers result in poor mechanical strength, which poses a significant challenge for industrial deployment.

The development of multichannel hollow fiber membranes (Figure 3b) reflects the breakthrough of performance bottlenecks through configuration innovation. Zhu et al. [34] successfully addressed the issue of low mechanical strength in hollow fiber membranes by developing novel four-channel hollow fiber oxygen-permeable membranes using the phase inversion method (Figure 3d). Building on this achievement, they proposed seven- [35] and nineteen-channel [36] hollow fiber membranes with multiple oxygen transport pathways. Additionally, hollow fiber perovskite membranes with corrugated surfaces have been developed and are considered promising as catalyst support [37]. Compared to smooth hollow fiber membranes without surface corrugations, the oxygen permeation flux of corrugated hollow fiber membranes demonstrates a remarkable threefold enhancement. Moreover, the structure and geometry of the membranes also influence temperature and gas flow distributions, which directly affect oxygen permeation rates.

The oxygen permeation mechanism in MIEC membranes fundamentally constitutes a multiscale coupled phenomenon driven by the synergistic transport of oxygen ions and electrons within the material matrix. This dynamic process is governed by the synergistic regulation of lattice oxygen vacancy concentrations, surface exchange kinetics, and bulk-phase diffusion capabilities. The oxygen transport mechanism can be systematically categorized into five consecutive steps [38,39,40,41]:

(1) Feed-side convection and diffusion: Oxygen molecules undergo physical adsorption onto membrane surfaces at the feed side through interactions with active surface sites, where adsorption efficiency is determined by binding energy and surface chemical characteristics.
(2) Feed-side surface exchange: Adsorbed oxygen molecules dissociate into atomic oxygen species via surface catalytic reactions. This energy-intensive process requires overcoming activation barriers, typically achieved through thermal activation or catalytic mediation.
(3) Bulk-phase diffusion: Dissociated oxygen atoms are incorporated into the crystal lattice as ionic species through coordination with metal cations, concurrently generating oxygen vacancies that serve as migration pathways. This step involves electron liberation and initiates oxygen ion migration through vacancy-mediated hopping mechanisms.
(4) Permeate-side surface exchange: Migrated oxygen ions recombine into molecular oxygen at the permeate side under partial pressure gradients. The desorption process necessitates overcoming specific energy thresholds to complete the net oxygen transfer across the membrane.
(5) Permeate-side convection and diffusion: Liberated oxygen molecules desorb from the membrane surface and diffuse into the low-oxygen partial pressure gas phase, completing the transmembrane transport cycle.

Mathematically, Wagner’s equation can often describe the oxygen permeation flux in MIEC membranes, as it considers the membrane’s thickness, the temperature, the oxygen partial pressure difference, and the material’s ionic and electronic conductivity. This relationship indicates that thinner membranes with higher conductivities can achieve more excellent oxygen permeation rates, making material selection and configuration crucial in optimizing MIEC membranes for industrial applications.

Several factors influence the oxygen permeation performance of MIEC membranes, including temperature, membrane thickness, and feed gas composition. Temperature plays a significant role: higher temperatures enhance ionic conductivity and oxygen surface exchange rates, thereby increasing oxygen flux. Membrane thickness is inversely proportional to the permeation flux: thinner membranes reduce the diffusion path, thus promoting higher oxygen permeation. However, mechanical stability and durability may be compromised in thinner membranes. The composition of the feed gas, especially the oxygen partial pressure, significantly affects the permeation rate. High oxygen partial pressures on the feed side and low partial pressures on the permeate side maximize the driving force for oxygen migration across the membrane.

## 3. CFD Modeling of MIEC Oxygen-Permeable Membranes

The modeling of MIEC oxygen-permeable membranes using CFD is essential for accurately simulating oxygen permeation, transport mechanisms, and reactor dynamics. The CFD modeling of MIEC membranes allows researchers to explore different process parameters, optimize membrane configurations, and analyze factors influencing oxygen flux and efficiency. In this section, we introduce the foundational mathematical models that describe oxygen permeation and then we delve into CFD applications, emphasizing the impact of flow patterns, boundary conditions, and computational settings.

### 3.1. Mathematical Models

The modeling of MIEC membranes involves mathematical equations describing the transport phenomena that occur within the membrane. These equations address the oxygen permeation flux, momentum conservation, energy balance, and component transport. Understanding these models is crucial for predicting the performance of MIEC membranes under various operating conditions.

#### 3.1.1. Oxygen Permeation Models

In existing studies, various computational methods have been employed to calculate the oxygen permeation flux of MIEC membranes. These methods exhibit diverse mathematical forms depending on the transport mechanism of the membrane, operating conditions, and model assumptions. Li et al. [42] summarized several common oxygen flux equations and their application scenarios. Among these, the models described below have been applied to CFD simulations.

Most researchers adopt the Wagner theory model and its modified versions [43]. These models primarily describe oxygen permeation processes under conditions such as thick membranes, single-phase MIEC membranes, and steady-state high-temperature operations, particularly when bulk diffusion dominates. The original theoretical model is expressed as follows:

(1) $$J_{O_{2}}=\frac{1}{4^{2}F^{2}L}\int_{\mu_{O_{2}\left(Ⅱ\right)}}^{\mu_{O_{2}\left(Ⅰ\right)}}t_{ion}t_{e}\sigma^{tot}d\mu_{O_{2}}$$

Here, $J_{O_{2}}\;$ is the oxygen permeation flux (mL·min^−1^·cm^−2^); F is the Faraday constant (C·mol^−1^); L is the membrane thickness (cm); $t_{ion}$ and $t_{e}$ are the ionic and electronic transference numbers, respectively; $\sigma^{tot}$ is the total conductivity (S·cm^−1^); and $\mu_{O_{2}}$ is the chemical potential of neutral oxygen in the oxide (J·mol^−1^).

**Figure 4:**
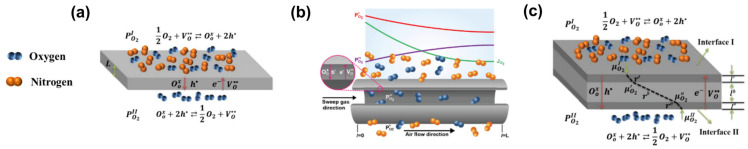
Schematic diagram of three oxygen permeation models: (**a**) Xu–Thomson model, (**b**) Tan and Li model, and (**c**) Zhu model. Reprinted with permission from Ref. [49]. Copyright 2017 John Wiley and Sons.

Xu and Thomson [44] were the first to establish a single-phase MIEC oxygen permeation model with a perovskite structure based on the surface reaction model proposed by Lin et al. [45] (Figure 4a). This model assumes that oxygen permeation flux is primarily controlled by oxygen vacancy diffusion and incorporates the effects of surface exchange reactions. It is particularly suitable for studies where variations in oxygen vacancy concentration depend on the oxygen partial pressure.

(2) $$J_{O_{2}}=\frac{k_{r}/k_{f}\left({P_{O_{2}}^{\prime \prime }}^{-1/2}-{P_{O_{2}}^{\prime }}^{-1/2}\right)}{\left(1/k_{f}{P_{O_{2}}^{\prime }}^{1/2}\right)+\left(2L/D_{v}\right)+\left(1/k_{f}{P_{O_{2}}^{\prime \prime }}^{1/2}\right)}$$

Here, $k_{r}$ is the reverse surface exchange rate constant, $k_{f}$ is the forward surface exchange rate constant, $D_{v}$ is the diffusion coefficient of oxygen vacancies, $P_{O_{2}}^{\prime }$ represents the oxygen partial pressure on the feed side (atm), and $P_{O_{2}}^{\prime \prime }$ denotes the oxygen partial pressure on the permeate side (atm).

The Kim model [46] simulates the oxygen permeation process in disc and tubular membranes, assuming that the oxygen vacancy density at the membrane interface does not significantly vary with the oxygen partial pressure. Different model equations have been proposed for the two membrane configurations:

The oxygen permeation model for disc membranes is as follows:

(3) $$\frac{{4nLJ}_{O_{2}}}{\langle c_{ion}D_{a}\rangle }=\ln\frac{\sqrt{P_{O_{2}}^{\prime }/P_{O_{2}}^{o}}-2J_{O_{2}}/c_{ion}^{\prime }k}{\sqrt{P_{O_{2}}^{\prime \prime }/P_{O_{2}}^{o}}-2J_{O_{2}}/c_{ion}^{\prime \prime }k}$$

The oxygen permeation model for tubular membranes is as follows:

(4) $$\frac{2nN_{O_{2}}}{\pi \langle c_{i}D_{a}\rangle w}\ln\frac{r_{1}}{r_{2}}=\ln\frac{{\left(P_{O_{2}}^{\prime }/P_{O_{2}}^{o}\right)}^{n}-N_{O_{2}}/\pi r_{1}wc_{i1}k}{{\left(P_{O_{2}}^{\prime \prime }/P_{O_{2}}^{o}\right)}^{n}+N_{O_{2}}/\pi r_{2}wc_{i2}k}$$

Here, n is the order of the chemical reaction at the gas–membrane interface, $N_{O_{2}}$ is the oxygen molar flow rate (mol·s^−1^), $c_{i}$ is the concentration of species *i* (mol·cm^−3^), $c_{ion}$ is the concentration of oxygen ions (mol·cm^−3^), $D_{a}$ is the diffusion coefficient of the oxygen ion–electron hole pairs (cm^2^·s^−1^), $P_{O_{2}}^{o}$ is the oxygen partial pressure at 1 atm of oxygen (atm), w is the length of the tubular membrane (cm), and $r_{1}$ and $r_{2}$ are the outer and inner radii of the tubular membrane (cm).

The Tan and Li model [47], building upon the Xu–Thomson model, establishes an oxygen permeation model specifically for hollow fiber MIEC membranes (Figure 4b). This model assumes that oxygen transport occurs in the radial direction, neglecting axial diffusion, and that gas-phase mass transfer resistance is minimal. As a result, the oxygen partial pressure at the membrane surface is considered equivalent to that on the gas side (either the lumen or shell side). These modeling assumptions are applicable to hollow fiber membranes with a high length-to-diameter ratio, where the length of the membrane is significantly greater than its diameter.

(5) $$J_{O_{2}}=\frac{k_{r}\left({P_{O_{2}}^{\prime }}^{1/2}-{P_{O_{2}}^{\prime \prime }}^{1/2}\right)}{\frac{2k_{f}\left(R_{o}-R_{\mathrm{in}}\right){P_{O_{2}}^{\prime }}^{1/2}{P_{O_{2}}^{\prime \prime }}^{1/2}}{D_{v}}+\frac{R_{m}{P_{O_{2}}^{\prime }}^{1/2}}{R_{in}}+\frac{R_{m}{P_{O_{2}}^{\prime \prime }}^{1/2}}{R_{o}}}$$

In this equation, $R_{m}$ represents the logarithmic mean radius of the hollow fiber membrane (m), defined as follows:

(6) $$R_{m}=\frac{R_{o}-R_{in}}{\ln\left(R_{o}/R_{in}\right)}$$

Here, $R_{o}$ and $R_{in}$ are the outer and inner radii of the hollow fiber membrane (m), respectively.

The Zhu model [48] is applied to analyze oxygen permeation in single- and dual-phase disc membranes, where both surface exchange reactions and bulk diffusion limit the process (Figure 4c). This model indicates that, for such MIEC membranes, the oxygen vacancy concentration is not strongly dependent on the oxygen partial pressure. Instead, the oxygen permeation flux is mainly determined by the resistance to oxygen transport within the membrane and the chemical potential difference:

(7) $$J_{O_{2}}=\frac{∆\mu_{O_{2}}^{tot}}{4^{2}F^{2}}\frac{1}{r_{tot}}$$

Here, $∆\mu_{O_{2}}^{tot}$ is the total chemical potential difference of oxygen molecules across the membrane (J·mol^−1^), and $r_{tot}$ is the total resistance of oxygen across the membrane (Ω·m^2^).

Liu et al. [50] simplified the complex transport processes by deriving the following empirical equation through experimental data fitting. This equation enables quick predictions of oxygen permeation performance, particularly for preliminary studies in large-scale industrial applications.

(8) $$J_{O_{2}}=Ak\left(T\right)G\left[{\left(P_{O_{2}}^{\prime }\right)}^{n}-{\left(P_{O_{2}}^{\prime \prime }\right)}^{n}\right]\;(n<1)$$

(9) $$J_{O_{2}}=Ak\left(T\right){\left[\frac{P_{O_{2}}^{\prime }}{P_{O_{2}}^{\prime \prime }}\right]}^{n}\;(n>0)$$

Parameters A  and n represent material properties and are obtained from curve fits of experimental data, $k\left(T\right)$ is a temperature factor (incorporating mobility), and G is a geometric factor.

In Table 1, we summarize the abovementioned oxygen permeation mathematical models that are applied to the CFD model, as well as their application scenarios.

#### 3.1.2. Mass Conservation

The continuity equation is one of the fundamental equations in fluid dynamics, describing the principle of mass conservation. It states that mass cannot be spontaneously created or destroyed within a control volume. Ensuring a uniform fluid distribution when designing MIEC oxygen-permeable membrane modules is critical for enhancing membrane performance. The continuity equation calculates the fluid distribution within complex geometries and identifies potential dead zones or regions of excessive flow. It is particularly important for optimizing the gas distribution within membrane reactors.

For incompressible fluids, the continuity equation is expressed as follows:

(10) $$\nabla \cdot \vec{u}=0$$

Here, $\vec{u}$ is the velocity vector (m·s^−1^). This indicates that the volumetric flow rate remains constant throughout the flow.

For compressible fluids, the continuity equation takes the following form:

(11) $$\frac{\partial \rho }{\partial t}+\nabla \cdot \left(\rho \vec{u}\right)=0$$

Here, ρ is the fluid density (kg·m^−3^), and t is the time (s). This formulation accounts for variations in density over time and space.

In CFD simulations, the numerical discretization of the continuity equation is typically performed using the finite volume method (FVM). The numerical solution ensures mass conservation by calculating the mass flux entering and exiting each control volume. Furthermore, to address the complexities of compressible flows, many CFD software packages employ pressure–velocity coupling algorithms, such as the SIMPLE or PISO algorithm, to satisfy the continuity and momentum conservation equations simultaneously.

#### 3.1.3. Momentum Conservation

The velocity distribution of the fluid directly affects the efficiency of oxygen transport across the membrane surface. Under laminar flow conditions, the flow is relatively smooth, and the dominant mechanism is the free diffusion of oxygen, which helps improve the uniformity of oxygen distribution. In contrast, increased velocity fluctuations and enhanced convective effects accelerate the oxygen transport rate to the membrane surface under turbulent conditions. The momentum conservation equation is fundamental to describing fluid flow behavior, based on the application of Newton’s second law, and it is expressed as follows:

(12) $$\rho \frac{\partial \vec{u}}{\partial t}+\rho \left(\vec{u}\cdot \nabla \right)\vec{u}=-\nabla P+\nabla \left(\mu \nabla \vec{u}\right)+\vec{F}$$

Here, P is the pressure (N·m^−3^), μ is the fluid viscosity (Pa·s or N·s·m^−2^), and $\vec{F}$ represents external forces (such as gravity or electromagnetic forces, N·m^−3^).

In membrane processes involving high flow rates, turbulent phenomena play a crucial role in performance. The commonly used turbulence models in membrane processes include the k-ε model (suitable for general turbulent flows, balancing computational efficiency and accuracy) and the k-ω model (more appropriate for low-Reynolds-number boundary layer flows, particularly effective in capturing near-wall flow behavior at the membrane surface). Researchers can optimize the geometric design of the membrane module and the fluid distribution system by numerically solving the momentum conservation equation. For instance, the impact of different inlet shapes (such as straight-through, diffusive, and jet flow shapes) on fluid distribution uniformity can be studied to minimize dead zones and pressure losses.

#### 3.1.4. Energy Balance

Temperature distribution directly affects the ionic and electronic conductivity of MIEC membranes. In high-temperature regions, the ionic mobility is enhanced, which increases the oxygen diffusion rate. However, temperature inhomogeneity within the membrane can lead to localized performance degradation and even material damage. Therefore, analyzing the thermal distribution through the energy equation can help optimize operating conditions. The energy equation incorporates convection, conduction, and radiative heat transfer, and it is coupled with reaction heat, typically expressed as follows:

(13) $$\rho C_{P}\frac{\partial T}{\partial t}+\rho C_{P}\left(\vec{u}\cdot \nabla T\right)=\nabla \cdot \left(k\nabla T\right)+S_{T}$$

Here, $C_{P}$ is the specific heat capacity (Jꞏkg^−1^·K^−1^), T is the temperature (K), k is the thermal conductivity (Wꞏm^−1^·K^−1^), and $S_{T}$ is the heat source term (Wꞏm^−3^). In steady-state simulations, the time-dependent term can be neglected ($\frac{\partial T}{\partial t}=0$).

The convective heat transfer driven by the gas flow ($\rho C_{P}\left(\vec{u}\cdot \nabla T\right)$) and the thermal conduction within the membrane ($\nabla \cdot \left(k\nabla T\right)$) jointly determine the temperature distribution of the membrane. The CFD model can optimize the synergy between convective and conductive heat transfer by adjusting the flow velocity, the direction, and the membrane’s geometric configuration.

#### 3.1.5. Component Transport

The diffusion of oxygen in MIEC membranes consists of surface exchange and bulk diffusion. Bulk diffusion is controlled by the ionic conductivity of the membrane material, while surface exchange is closely related to the adsorption characteristics of the membrane surface. By setting different diffusion coefficients, the transport characteristics of various membrane materials can be simulated. The process can be described using the component transport equation:

(14) $$\frac{\partial C}{\partial t}+\vec{u}\cdot \nabla C=\nabla \cdot \left(D\nabla C\right)+r$$

Here, C is the oxygen concentration (molꞏm^−3^), D is the diffusion coefficient (m^2^ꞏs^−1^), and r is the reaction term (molꞏm^−3^·s^−1^).

In certain oxygen-permeable membrane reactors, oxygen is the transported component, and it participates in chemical reactions (such as the partial oxidation of methane). In such cases, the reaction term r includes the consumption or production rate of oxygen. For example, the reaction rate for methane partial oxidation to syngas is commonly described using the Arrhenius equation:

(15) $$r=kC_{O_{2}}^{n}C_{{CH}_{4}}^{m}=Ae^{\frac{-Ea}{RT}}C_{O_{2}}^{n}C_{{CH}_{4}}^{m}$$

Here, k is the reaction rate constant (related to the reaction order, mol^1−n−m^ꞏm^3(n+m−1)^ꞏs^−1^), $C_{i}$ is the concentration of the reactants (molꞏm^−3^), n and m are the reaction orders, A is the pre-exponential factor (which has the same dimensions as k), Ea is the activation energy (Jꞏmol^−1^), and R is the gas constant (Jꞏmol^−1^ꞏK^−1^).

### 3.2. Geometric Models

**Figure 5:**
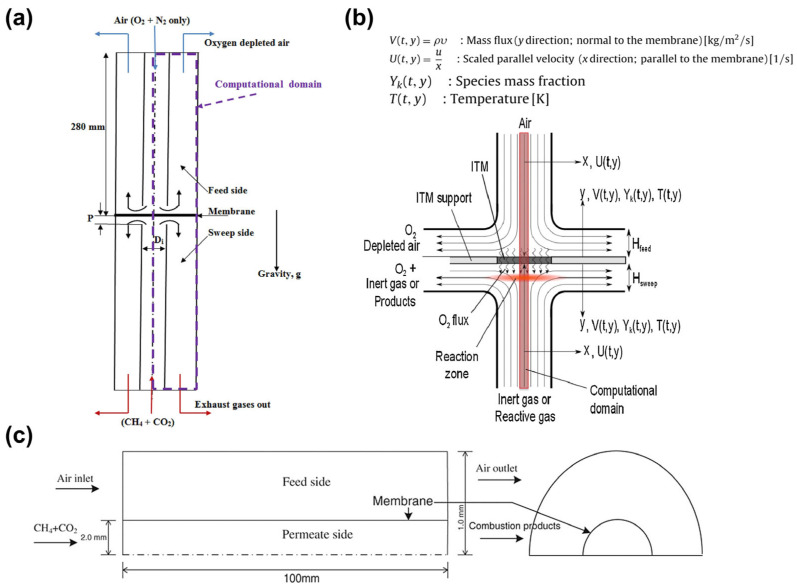
Schematic diagram of geometric models: (**a**) button-cell model, reprinted with permission from Ref. [51], copyright 2015 Elsevier; (**b**) finite-gap stagnation flow model, reprinted with permission from Ref. [53], copyright 2012 Elsevier; and (**c**) two-dimensional axisymmetric tube model, reprinted with permission from Ref. [68], copyright 2013 John Wiley and Sons.

Current CFD research on MIEC disk-shaped membrane configurations mainly falls into two categories. The first category involves the use of a button-cell model [51] (Figure 5a), which is commonly used in laboratory-scale experiments. In this simplified model, the focus is typically on examining the radial gas concentration changes across the membrane. The oxygen concentration distribution on the membrane surface is influenced by the diameters of the feed tubes on both sides, the distance between the feed tubes and the membrane, and the sweep gas flow rate [52]. This simplified model is beneficial for exploring the optimal geometric setup and operational conditions to achieve the maximum oxygen permeation flux.

The second category is the finite-gap stagnation flow model [53] (Figure 5b). This model simplifies analysis by reducing the model’s dimensions, allowing for more complex models to be investigated within limited computational resources. This simplification is based on the fact that, near the stagnation line, the distributions of flow variables such as velocity, temperature, density, and component concentration follow a self-similar solution in the direction perpendicular to the membrane. All normalized flow variables are functions of the membrane-normal direction and time, assuming boundary layer approximation, where oxygen diffusion along the x-direction is negligible compared to that along the y-direction. Therefore, when introducing more complex reactions on the permeate side, such as fuel combustion [54,55], partial oxidation [56,57,58,59,60], and oxidative coupling [61,62,63,64,65,66], this model is advantageous for examining the effects of detailed gas-phase chemistry and component transport on fuel conversion and mass transfer phenomena within the membrane reactor. In the stagnation flow model, the oxygen permeation flux on the membrane is uniformly distributed [67], which is unlikely to occur in the button-cell model or tubular, hollow fiber membrane models.

In tubular membrane simulations, the model is typically simplified to a two-dimensional axisymmetric model [68] (Figure 5c). This model is useful for examining the effects of operational modes [69] or feed methods [70] on oxygen permeation performance. This configuration is often used in reactor studies, where reactions occur on the permeate side. It provides important insights into the reactor’s temperature distribution and combustion characteristics, and it can guide the design of novel isothermal reactors.

**Figure 6:**
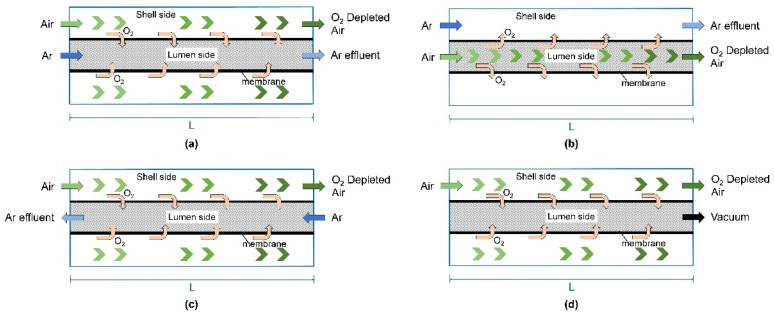
Four operating modes for oxygen permeation in hollow fiber membranes: (**a**) shell feed, co-current flow operation; (**b**) lumen feed, co-current flow operation; (**c**) shell feed, counter-current flow operation; (**d**) and shell feed, co-current flow with vacuum operation.

In CFD studies of hollow fiber membranes, researchers primarily employ three-dimensional models, though some also use two-dimensional axisymmetric models for simplified simulations. On the one hand, three-dimensional models are used to design and optimize membrane modules, as they minimize errors across all spatial directions. On the other hand, two-dimensional axisymmetric models reduce computational complexity while retaining key geometric features and physical mechanisms, enabling effective analyses of oxygen permeation performance. By simulating various operating modes, these models allow for in-depth explorations of the effects of different parameters on membrane oxygen permeation. Tan et al. [69] proposed four operating modes suitable for hollow fiber membranes (Figure 6) and compared vacuum operation on the permeate side with sweep gas purging. Their findings provide valuable guidance for scaling up hollow fiber membrane modules in future applications.

## 4. Application of CFD in MIEC Oxygen-Permeable Membranes

The application of CFD in studying MIEC oxygen-permeable membranes has significantly advanced our understanding of their complex behavior and operational efficiency. By providing detailed quantitative insights into transport phenomena and reaction kinetics, CFD has become an indispensable tool for optimizing membrane performance. This section explores the various applications of CFD in MIEC membranes, focusing on its contributions to analyzing heat and mass transfer processes and in guiding reactor and module design. These research areas address key challenges and opportunities in enhancing the practicality and scalability of MIEC membranes. The following subsections delve into the specific roles of CFD, demonstrating how these targeted studies provide predictive capabilities for performance optimization and offer practical strategies to overcome current design limitations.

### 4.1. CFD Studies of Heat and Mass Transfer Phenomena

In MIEC oxygen-permeable membrane systems, heat and mass transfer phenomena are crucial for optimizing membrane performance, especially when scaling membrane reactors from laboratory to industrial applications. This process is crucial for the functionality of MIEC membranes, involving understanding fluid dynamics at the membrane surface and the specific transport of oxygen within the membrane matrix. Navier–Stokes equations are typically used to capture these fluid dynamics. Coroneo et al. [71,72] developed specialized CFD models that accurately simulate oxygen transport within MIEC membranes. By incorporating source terms (*S_i_*), these models allow for the setup of oxygen-consuming sinks on the feed side and oxygen-generating sources on the permeate side (Equation (16)). This mechanism forms the foundational model for analyzing oxygen permeation within the CFD framework.

(16) $$S_{i}=\left\{\begin{array}{ll} +\frac{J_{O_{2}}\cdot A_{cell}}{V_{cell}} & \;\;\mathrm{At}\;\mathrm{permeate}\;\mathrm{side}\\ -\frac{J_{O_{2}}\cdot A_{cell}}{V_{cell}} & \;\;\mathrm{At}\;\mathrm{feed}\;\mathrm{side}\;\;\;\;\;\;\;\;\;\; \end{array}\right.$$

Here, $A_{cell}$ is the cell mass transfer area and $V_{cell}$ is the cell volume.

To further refine the simulation of oxygen transport, Serra et al. [52,73] used a multiphysics CFD approach combined with COMSOL Multiphysics v4.4. software to conduct an in-depth study of the flow characteristics and transport mechanisms in planar MIEC oxygen-permeable membranes. This method enabled them to simulate the interaction between feed-side conditions and oxygen permeation, identifying key factors that enhance oxygen flux, such as increasing the sweep gas velocity and reducing the spacing between membrane elements. Their findings indicated that smaller spacings and higher sweep gas velocities can improve oxygen flux but may also lead to polarization effects, where concentration gradients limit overall uniformity.

Xue et al. [74] also developed a multiphysics transport model using COMSOL Multiphysics, based on experimental setups, to numerically investigate oxygen permeation performance and the underlying mechanisms. The CFD model accounted for key physical and chemical processes, including gas flow, surface reactions, and ion and electron transport. The results showed that increasing the inlet pressure and reducing the permeate-side pressure significantly enhanced the oxygen permeation flux. Additionally, parameters such as the effective length, structural thickness, and temperature of the hollow fiber membrane played a crucial role in permeation behavior. For instance, thinner membrane layers reduced charge transport resistance and improved oxygen permeation efficiency, while thicker support layers increased gas diffusion resistance. Furthermore, the study revealed that the oxygen partial pressure on the permeate side greatly influenced the distribution of the oxygen vacancy concentration. Operating conditions can be adjusted to optimize the oxygen vacancy concentration and improve permeation performance. Increasing the operating temperature significantly enhances oxygen permeation efficiency due to the thermally activated nature of the process.

Ahmed et al. [75] conducted CFD simulations focused on optimizing sweep gas parameters, developing a computational model to predict the oxygen separation performance of La_2_NiO_4_ disc-shaped membranes. By varying the diameter of the sweep gas pipe and its distance from the membrane surface, their model revealed that a pipe diameter of 2 mm and a membrane spacing of 1 mm achieved the highest oxygen flux within existing geometric constraints. This study underscored the practicality of CFD in determining the optimal sweep gas size and positioning to maximize oxygen permeation efficiency. Additionally, in a subsequent study [76], they investigated the impact of sweep gas composition on the oxygen permeation performance. They conducted a detailed analysis of oxygen transport behavior in a button-cell model by introducing different sweep gas components on the permeate side. The researchers found that increasing the CH_4_ concentration in the feed resulted in a higher oxygen flux, which could be attributed to CH_4_ having a lower molecular weight than CO_2_. As the CH_4_ concentration rose, the volumetric flow rate of oxygen on the permeate side increased, reducing the oxygen partial pressure on the sweep side and thereby enhancing the driving force for oxygen transport.

**Figure 7:**
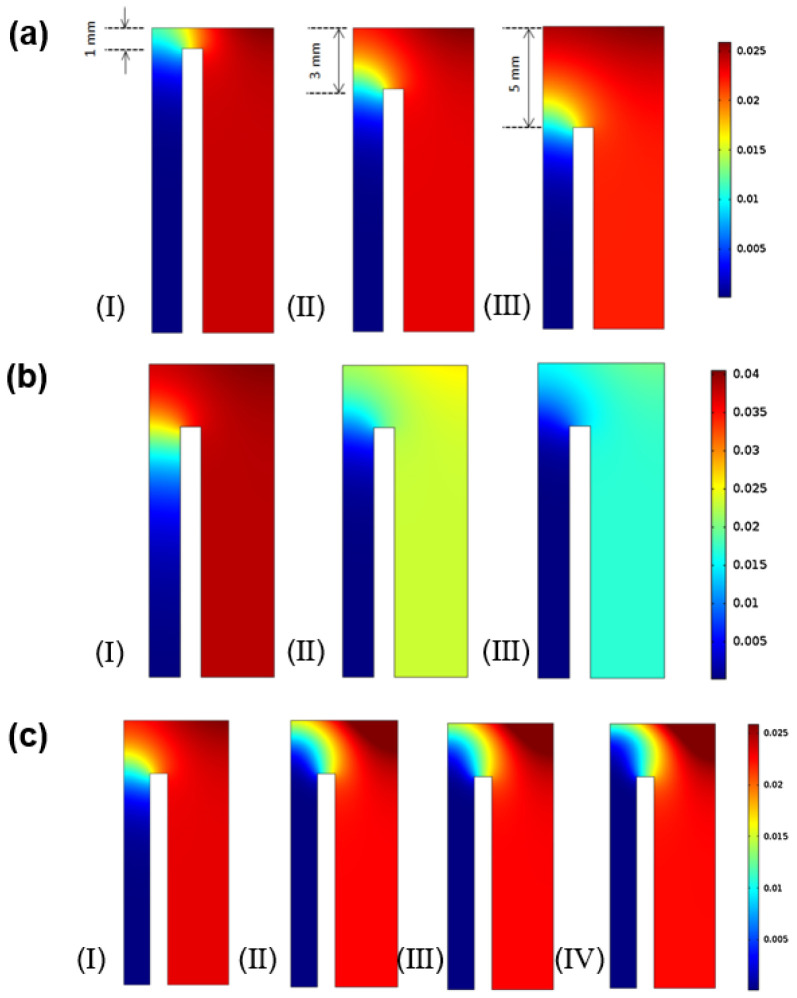
Contour of $P_{O_{2}}$ on the permeate side under different operating conditions: (**a**) D (distance from the sweep gas inlet to the membrane surface), where I, II, and III are D = 1, 3, and 5 mm, respectively, and helium is used as the sweep gas; (**b**) helium flow rate, where I, II, and III are helium flow rates of 30, 60, and 90 mL·min^−1^, respectively; and (**c**) type of sweep gas, where I, II, III, and IV are helium, nitrogen, argon, and carbon dioxide (60 mL·min^−1^), respectively. Reprinted with permission from Ref. [77]. Copyright 2015 Elsevier.

In MIEC oxygen-permeable membranes, ionic conductivity is a key parameter that describes the membrane material’s ionic conduction performance during oxygen permeation. Traditional oxygen permeation experiments typically use permeation setups to introduce the inlet and sweep gases directly onto the membrane surface. However, due to the presence of concentration gradients, the oxygen partial pressure at the outlet of the experimental setup does not align with that at the membrane surface, leading to inaccuracies in calculating the ionic conductivity. Chen et al. [77] proposed using a CFD model to precisely determine the oxygen partial pressure at the membrane surface. Based on this model, they studied the effects of various parameters, such as the distance from the sweep gas inlet to the membrane surface (Figure 7a), the sweep gas flow rate (Figure 7b), and the sweep gas type (Figure 7c), on the oxygen distribution at the membrane surface. As the sweep gas inlet distance from the membrane surface increased, the oxygen partial pressure distribution became more uniform; however, too large a distance resulted in a significantly higher oxygen partial pressure at the membrane surface than at the outlet. While a higher sweep gas velocity increases oxygen flux, it reduces the uniformity of the oxygen partial pressure. When the diffusion coefficient of the oxygen/sweep gas mixtures was higher, it helped accelerate oxygen transfer from the membrane surface, improving oxygen distribution uniformity and optimizing overall membrane performance. Simulation results also showed that, within the tested temperature and oxygen partial pressure ranges, the ionic conductivity values calculated using traditional assumptions were 50% to 180% higher than those obtained from the CFD model.

However, there are some limitations to their research. The study focused on SrCo_0.8_Fe_0.2_O_3−δ_ (SCF) membrane material but did not verify the applicability of the method to other MIEC materials. Different materials may have different physicochemical properties, which may affect the universality of the model. Moreover, they did not compare the CFD method with other possible methods for measuring ionic conductivity, such as direct measurement techniques or other simulation methods. Such comparisons would help directly assess the strengths and weaknesses of the CFD method.

The practical application of ceramic oxides at high temperatures often requires the tuning of the thermal expansion coefficient to achieve thermal expansion compatibility among different oxides. The thermal expansion coefficient determines the extent of lattice expansion as the material heats up. In oxygen-deficient perovskite oxides, lattice expansion is primarily driven by two mechanisms: (1) physical expansion, which is caused by an increasing temperature, and (2) chemical expansion, which results from an increase in oxygen non-stoichiometry and a partial reduction in B-site metal ions under high temperatures and low oxygen partial pressures [78,79,80,81,82]. For the industrial application of MIEC oxygen-permeable membranes, chemical expansion is particularly significant. One side of the membrane is exposed to a low oxygen partial pressure, causing chemical expansion, while the other is in contact with an air atmosphere. This disparity induces substantial mechanical stress within the membrane, which can lead to cracking. Therefore, minimizing chemical expansion is a critical goal in industrial applications.

Zolochevsky et al. [83] developed a mathematical model linking chemically induced strain and stress to oxygen non-stoichiometry in membrane materials. Using ANSYS 11.0 for numerical simulations, they investigated how the interaction between oxygen surface exchange kinetics and membrane thickness influences the stress distribution and oxygen permeation flux. For membranes with a thickness significantly larger than the characteristic thickness, the calculated stress under the assumption of constant oxygen non-stoichiometry closely matched the results considering surface exchange kinetics. However, for thinner membranes (approaching the characteristic thickness), surface exchange kinetics significantly reduced chemically induced stress, particularly the maximum von Mises stress on the permeate side, with reductions of up to fourfold. Furthermore, the study demonstrated that decreasing the membrane thickness notably increased oxygen permeation flux. Nevertheless, ignoring surface exchange kinetics in modeling may lead to an overestimation of stress and an underestimation of membrane durability. This highlights the importance of incorporating accurate surface exchange kinetics in the analysis, especially for thinner membranes or those close to the characteristic thickness.

In laboratory-scale MIEC planar membrane reactors, the design of the sweep gas inlet enables a portion of the fuel to escape from the reaction zone. This helps maintain a constant temperature on the membrane surface and prevents membrane rupture due to thermal stress. However, for tubular, hollow fiber, and pilot-scale MIEC membrane reactors, the non-uniform distribution of oxygen permeation flux along the membrane length can lead to uncontrolled combustion of the sweep-side fuel and heat release. In such reactors, significant temperature gradients can induce wall shear stress, which inevitably causes membrane damage and limits the application potential of MIEC membrane reactors. To address these challenges, many researchers use CFD technology to optimize the operational parameters of these membrane reactors and to guide the design of more efficient isothermal membrane reactors.

Bai et al. [84] used a CFD model to investigate how fluid dynamics and combustion reactions influence oxygen permeation, heat, and mass transfer in Ce_0.85_Sm_0.15_O_2−δ_-Sm_0.6_Sr_0.4_Al_0.3_Fe_0.7_O_3−δ_(SDC-SSAF) dual-phase oxygen transport membranes. Their results indicated that increasing the feed-side pressure or reducing the permeate-side vacuum enhanced the oxygen partial pressure gradient, significantly boosting oxygen permeation rates. Additionally, introducing CH_4_ combustion on the permeate side raised the membrane’s operating temperature, accelerating oxygen transport and reducing oxygen accumulation on the permeate-side edges, further improving permeation efficiency. Under combustion conditions, oxygen permeability at 800 °C was enhanced by 190.92% compared to under non-combustion conditions. Additionally, geometric parameters were found to be crucial for membrane performance; a 3 mm inlet gap achieved optimal oxygen permeation, offering valuable guidance for membrane system design. However, in their research, the treatment of the methane combustion reaction was simplified, considering only a single-step finite-rate reaction kinetics model. The actual combustion process may involve more complex reaction steps and intermediate products, which could affect the accuracy of the model.

**Figure 8:**
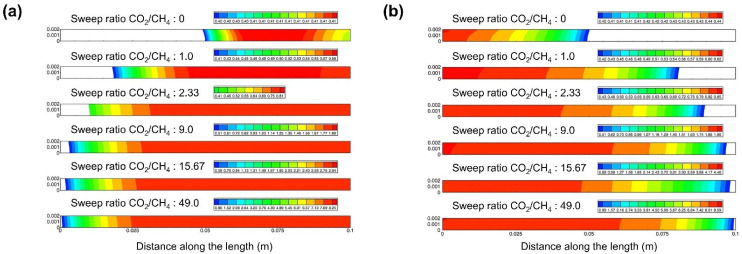
Oxygen-to-methane mass fraction ratio for different inlet conditions of sweep ratio (CO_2_/CH_4_) in (**a**) co-current flow operation and (**b**) counter-current flow operation. Reprinted with permission from Ref. [70]. Copyright 2012 John Wiley and Sons.

Farooqui et al. [70] conducted a CFD study on the impact of the permeation-side fuel ratio (CO_2_/CH_4_) on fuel combustion (O_2_/CH_4_) in tubular membranes under co-current (Figure 8a) and counter-current (Figure 8b) operations. The simulation results indicated that a CO_2_/CH_4_ ratio of approximately 24 under co-current conditions and about 15.67 under counter-current conditions could achieve stoichiometric combustion along the membrane length. This minimized fuel-rich and fuel-lean regions that create thermal gradients along the membrane, thus ensuring optimal reactor performance. Additionally, they found that counter-current operation led to a more uniform oxygen flux distribution along the reactor walls and improved the mixing of fuel and oxygen.

Ben-Mansour et al. [85] further optimized the design of isothermal reactors using CFD, focusing on minimizing the mass flow rate of the sweep gas to maintain a uniform temperature distribution. Their simulations demonstrated that this approach could achieve near-isothermal conditions over most of the membrane length, thereby enhancing CH_4_ conversion rates. In contrast, higher sweep gas flow rates significantly reduced CH_4_ conversion efficiency. In response to these findings, they proposed dividing the membrane reactor into a series of smaller units with segmented fuel inlets to mitigate uneven thermal distributions and improve overall thermal management.

**Figure 9:**
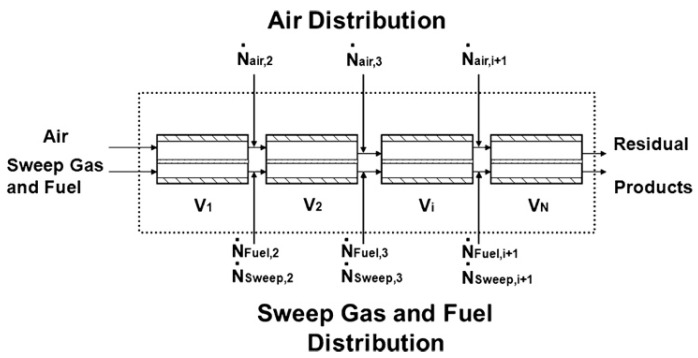
Schematic diagram of MCRI. Reprinted with permission from Ref. [86]. Copyright 2012 American Chemical Society.

Mancini et al. [86] validated this segmented reactor concept in their study by designing a multiple-compartment reactive ion transport (MCRI) membrane reactor (Figure 9). This approach divided the entire MIEC membrane reactor into sequentially connected inlet flow units. The design effectively addressed the critical issues of the low fuel conversion rates and non-uniform temperature distribution within the reactor, making thermal management more efficient and achieving higher average oxygen permeation flux. Compared to conventional MIEC membrane reactors, MCRI significantly reduced energy consumption and the amount of membrane material required, demonstrating clear advantages.

**Figure 10:**
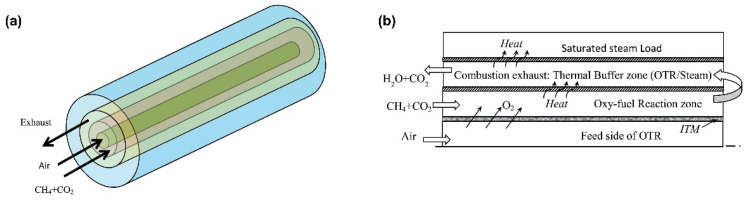
Schematic diagram of the two-pass fire-tube boiler coupled with OTR: (**a**) three-dimensional model and (**b**) axisymmetric cross-section model. Reprinted with permission from Ref. [87]. Copyright 2016 John Wiley and Sons.

Using pure oxygen instead of air significantly reduces nitrogen oxide emissions and facilitates CO_2_ capture in oxy-fuel combustion, enabling low-carbon or zero emissions. In a separate study, Ben-Mansour et al. [87] integrated membrane reactors into fire-tube boilers to investigate the oxygen transport reactor (OTR) under high temperatures (Figure 10). They focused on the oxygen permeation efficiency, full oxygen combustion characteristics in the fire-tube boilers, and heat transfer effects. The results showed that operating the system at high temperatures of around 1373 K in the upstream region of the reactor substantially increased oxygen permeation and combustion reaction rates, generating high localized heat fluxes that enhanced steam generation efficiency in the boiler. At high methane concentrations, combustion was concentrated near the inlet, which helped optimize the thermal output and reduce heat loss. When the methane concentration in the fuel mixture was raised to 6%, the reaction rate and oxygen permeation significantly improved; however, further increases in the methane concentration had a limited impact, likely due to the rapid oxygen depletion limiting sustained reactions. Furthermore, it was observed that adjustments in the fuel flow rate had minimal impact on the overall performance of the reactor.

Mansir et al. [88] further explored the geometric design and heat transfer characteristics of a dual-channel oxygen transport reactor within fire-tube boilers, using numerical simulations to analyze how parameters such as gas inlet temperature and mass flow rate affect oxygen permeation and combustion stability. They found that, under non-reactive conditions (with CO_2_ as the sweep gas), the inlet temperature and mass flow rate had minimal effect on oxygen permeation. However, under reactive conditions (with CH_4_ and CO_2_ as the sweep gases), these parameters had a significant impact on the oxygen permeation rate and overall system performance. They also highlighted the importance of gas inlet conditions, particularly sweep gas inlet conditions, for combustion stability [89]. This contrasts with Ben-Mansour’s conclusion that fuel flow rate variations did not notably affect reactor performance, likely because Ben-Mansour’s experimental design emphasized temperature and fuel concentration as the primary controls on oxygen permeation and combustion rate rather than flow rate changes. Mansir’s OTR design can accommodate power outputs between 1 and 5 MWe, representing an important advancement for fire-tube boiler applications. By optimizing these parameters, oxy-fuel combustion technology can be more effectively applied to fire-tube boilers, enhancing energy efficiency and reducing emissions.

Additionally, these conclusions were validated in a study by Zhao et al. [90], who conducted simulation analyses on syngas combustion in fire-tube boiler applications using CO, H_2_, and CO_2_ as the sweep gases. Their results similarly demonstrated that high-temperature operation, an increased sweep gas flow rate, and a higher H_2_/CO mass ratio in the syngas enhanced oxygen permeation through the membrane and improved heat transfer efficiency, thus enhancing membrane reactor performance. The inlet temperature of the reactor had a significant effect on oxygen permeation and heat generation. When the temperature increased from 1073 K to 1273 K, the oxygen permeation rate surged to 34 times its original value, and the thermal power output nearly doubled. At low sweep gas flow rates, changes in the membrane temperature and radiative heat exchange were minimal. However, as the flow rate increased, oxygen permeation and convective heat exchange rose substantially. Moreover, as the H_2_/CO mass ratio increased, the proportion of radiative heat transfer gradually decreased. In contrast, convective heat transfer increased, likely due to the radiative properties of water vapor from H_2_ combustion being lower than those of CO_2_ from CO combustion.

### 4.2. CFD Studies of Membrane Reactor Design

In the previous section, we investigated the fundamental heat and mass transfer phenomena, which are crucial for understanding membrane behavior at the microscale. Building on this foundation, we extend the discussion to the practical application of these principles in the design of membrane reactors. By analyzing how microscale heat and mass transfer processes collectively impact the performance of individual reactors, we bridge the gap between theoretical understanding and practical engineering solutions, demonstrating the key role of CFD in optimizing membranes and their integrated reactor systems.

Compared to the pure oxygen separation process in MIEC oxygen-permeable membranes, MIEC membrane reactors must simultaneously meet the dual requirements of air separation and fuel conversion, which involve complex chemical reactions on the permeate side. CFD has become an essential tool for optimizing these reactors to achieve diverse configurations and performance targets. Early studies on membrane reactors neglected temperature variations caused by chemical reactions [55,91], incorporated detailed reaction models but overlooked mass and heat transfer processes from the gas phase to the membrane surface [59,92], or expressed oxygen permeation rates based on bulk flow parameters instead of the local thermodynamic state at the membrane surface [60]. These approaches all have certain limitations. Most researchers studying combustion performance via CFD have focused primarily on homogeneous chemical reactions on the permeate side, often neglecting the interaction between these reactions and the membrane surface. Therefore, developing modeling methods capable of spatially resolving the thermochemical field near the membrane is crucial.

**Figure 11:**
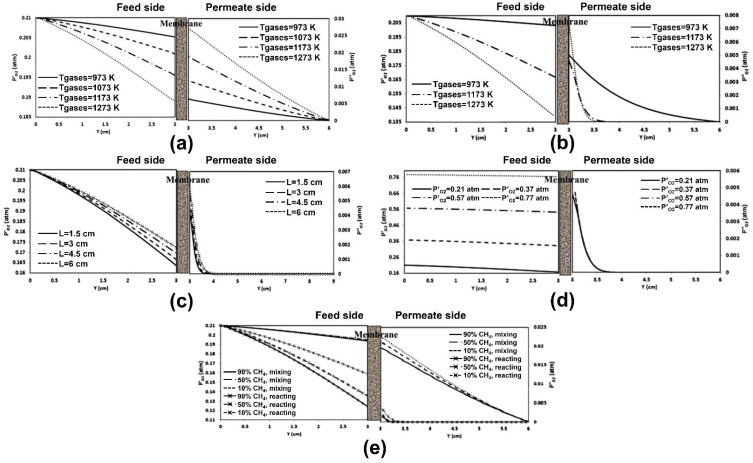
Oxygen partial pressure varies with different operating conditions: (**a**) feed and sweep gas inlet temperature (without reactions), (**b**) feed and sweep gas inlet temperature (with reactions), (**c**) normal distance to the membrane (L), (**d**) oxygen partial pressure on the feed side, and (**e**) percentages of CH_4_/CO_2_ as sweep gases. Reprinted with permission from Ref. [98], copyright 2013 Elsevier.

Habib et al. [93] were the first to conduct numerical studies on methane combustion characteristics within membrane reactors under the stagnation flow model. Due to the high computational demands of simulating combustion reactions, they used a simplified reaction mechanism to reduce computational costs, assuming that the chemical reaction products were only carbon dioxide and water. They also applied the “fast kinetics” assumption (equilibrium limit), where the permeated oxygen immediately reacted with the fuel and was consumed, meaning that the transmembrane oxygen transfer primarily limited the reaction rate. However, their model had limitations, as its predictive accuracy was affected by changes in the fuel composition on the permeate side [94]. Therefore, detailed studies using comprehensive multi-step reaction mechanisms were necessary [95,96]. In their subsequent research [97], they employed a two-step oxygen combustion reaction kinetics model to investigate the combustion characteristics and oxygen permeation performance within MIEC membrane reactors. The simulation results closely matched various experimental datasets, enabling a more precise analysis of multiple factors that influence membrane reactor performance. The results indicated that parameters such as inlet gas temperature, CH_4_ content in the sweep gas, and reactor geometry significantly impacted the operation of the MIEC membrane reactor, while the effects of the feed and sweep gas volumetric flow rates, as well as the oxygen partial pressure on the feed side, were relatively minor. Moreover, they examined oxygen permeation under various conditions, including non-reactive conditions using an inert gas (Ar) as the sweep gas and reactive conditions using a CH_4_ and CO_2_ gas mixture as the sweep gas [98] (Figure 11).

**Figure 12:**
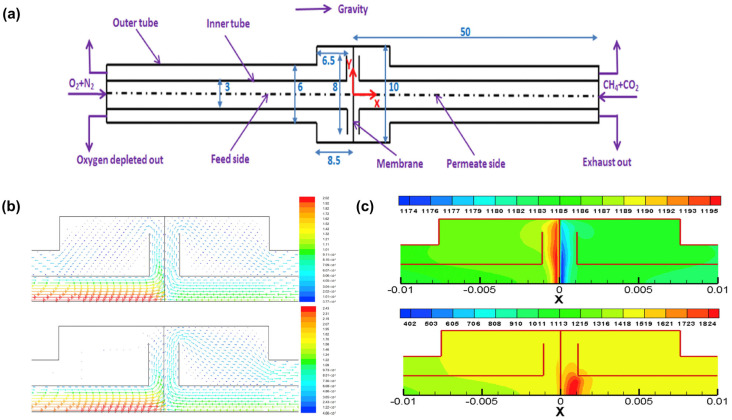
(**a**) Schematic diagram of the modified design of a button-cell membrane reactor (all dimensions are in mm). Comparison between non-reactive (upper plot) and reactive (lower plot) flows in terms of (**b**) velocity vectors and (**c**) temperature contours. Reprinted with permission from Ref. [99]. Copyright 2016 Elsevier.

Additionally, utilizing a modified oxygen permeation model, they investigated the characteristics of methane oxy-fuel combustion in a modified button-cell membrane reactor [99] (Figure 12a). The study focused on the impact of flow conditions on both sides of the membrane, including variations in Reynolds number and secondary surface reactions. Stable combustion was achieved through the formation of low-velocity and recirculation zones near the membrane (Figure 12b). The results showed that oxygen permeation rates under reactive flow conditions were significantly higher than those under non-reactive flow conditions, primarily due to the increased membrane surface temperature caused by combustion (Figure 12c). As the inlet temperature increased, the oxygen permeation rate under non-reactive flow conditions rose markedly, whereas that under reactive flow conditions was primarily governed by flame temperature. Increasing the fuel concentration, sweep gas flow rate, and oxygen partial pressure on the feed side effectively enhanced oxygen permeation rates and combustion performance. However, care must be taken to avoid overheating the membrane surface, which could damage the material.

**Figure 13:**
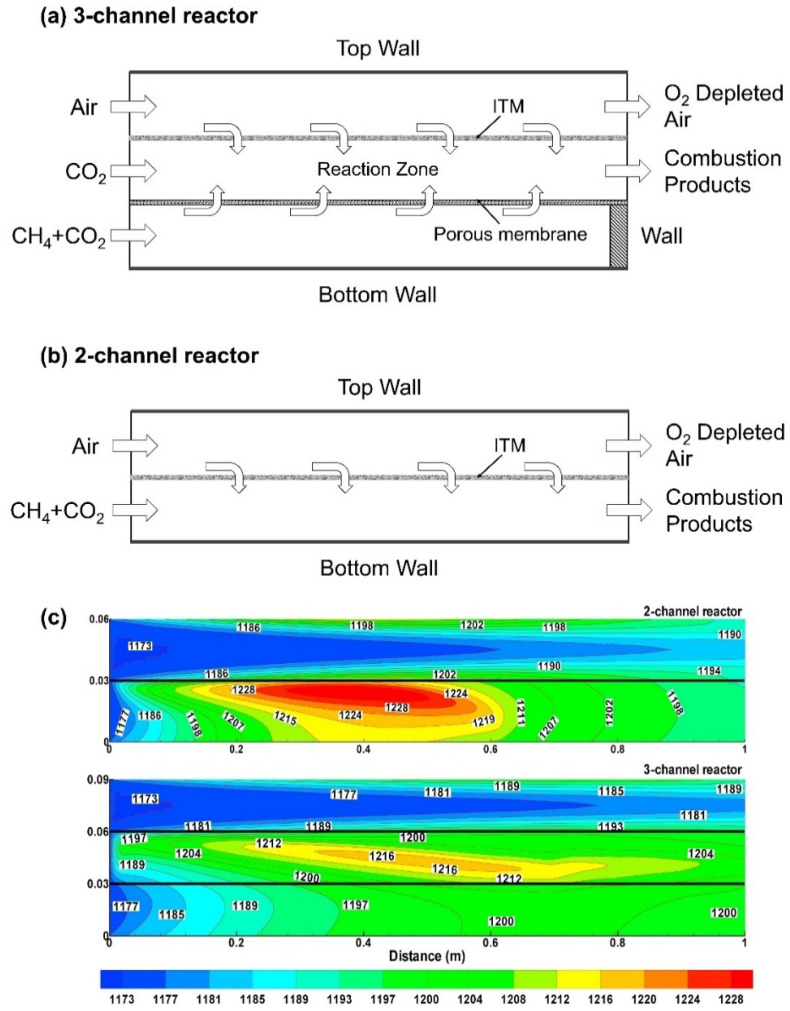
Schematic diagram of (**a**) the 3-channel reactor and (**b**) the 2-channel reactor. (**c**) Comparison of contours of temperature for 2- and 3-channel reactors for a mass flow rate of fuel mixture $M_{f}=0.0015\;kgꞏs^{-1}$ and ${CH}_{4}=1\%$. Reprinted with permission from Ref. [100]. Copyright 2014 Elsevier.

The traditional two-channel MIEC membrane reactor used in oxy-fuel combustion is prone to high temperature gradients and localized hot spots, which can lead to membrane damage. To address this, Ahmed et al. [100] applied CFD simulations to develop a three-channel membrane reactor (Figure 13a) and compared its performance with that of the traditional two-channel reactor (Figure 13b). In the three-channel structure, the porous plate was divided into 10 equally spaced sections with varying porosity to effectively regulate fuel distribution on the permeate side, achieving a more uniform combustion process. The simulation results indicated that high-temperature regions in the two-channel reactor were concentrated near the inlet and membrane surface. In contrast, in the three-channel reactor, the reaction zone extended along the entire membrane length, resulting in a more uniform temperature distribution (Figure 13c). This uniform thermal flux reduced the shear stress on the membrane surface caused by high temperature differentials, enhancing membrane stability and lifespan. Additionally, the three-channel reactor exhibited higher exit temperatures, significantly improving the thermodynamic efficiency of the MIEC membrane reactor.

Habib et al. [101] also conducted numerical modeling of an isothermal reactor that combines MIEC membranes with a porous membrane to predict its oxy-combustion characteristics. They applied porous jump boundary conditions to simulate a thin “porous membrane” with known velocity (pressure drop) characteristics. This model optimized the resistance characteristics of porous membranes, facilitating oxygen separation while promoting uniform combustion and preventing the formation of high-temperature zones commonly seen in conventional dual-channel reactors. Although this was achieved in a simplified one-dimensional model of a porous medium, further research is required to determine whether it can be achieved in multidimensional models. The simulation results demonstrated that the isothermal reactor could achieve approximately uniform combustion along the membrane length. The model was validated, showing good agreement between the simulation results and experimental data reported in the literature. However, the range of the experimental data may be limited, covering only specific operating conditions and parameter ranges. The applicability and accuracy of the model under a wider range of conditions need further verification. Future research will optimize the three-channel design by considering factors such as fuel cracking, channel height, feed flow rate, and porous-plate structure.

Nemitallah et al. [102] used a porous-plate reactor (PPR) to simulate the oxygen combustion characteristics in an OTR, investigating combustion efficiency, temperature distribution, and flame stability through experiments and three-dimensional numerical simulations. The results showed that the porous plate effectively distributed oxygen and stabilized the flame, preventing high temperatures from damaging the membrane while enhancing oxygen permeation efficiency. As the CO_2_ concentration in the oxidizer increased, the flame became more elongated, and the position of complete combustion shifted further downstream. Under stoichiometric conditions, the excellent mixing effect and flame stability enabled complete fuel combustion. Additionally, the addition of CO_2_ resulted in the formation of an outer recirculation zone (ORZ), which improved flame stability, optimized the thermal environment around the porous plate, and facilitated enhanced oxygen permeation. Building on their research, Tahir et al. [103] investigated the differences in combustion behavior between vertical and horizontal PPRs, focusing on the effects of the inlet flow rate, oxidizer ratio, and inlet temperature on the temperature distribution and methane conversion efficiency. The study revealed that vertical reactors achieved a more uniform temperature distribution than horizontal reactors, significantly reducing the risk of high-temperature hotspots and extending the equipment’s operational lifespan.

**Figure 14:**
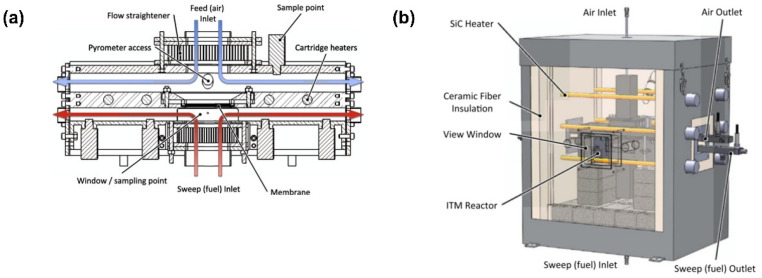
Schematic diagram of an ITM reactor: (**a**) cross-section view and (**b**) overall view of the installation inside the furnace. Reprinted with permission from Ref. [104]. Copyright 2013 Elsevier.

Kirchen et al. [104] developed a novel ion transport membrane (ITM) reactor based on the planar stagnant flow model (Figure 14). This reactor employed a “probe” optical measurement technique to monitor the membrane temperature, enabling the cross-verification of fuel conversion and oxygen permeation behavior through spatially resolved numerical simulations. They examined the differences between the mixed gas composition measured at the reactor outlet and that measured near the membrane surface.

**Figure 15:**
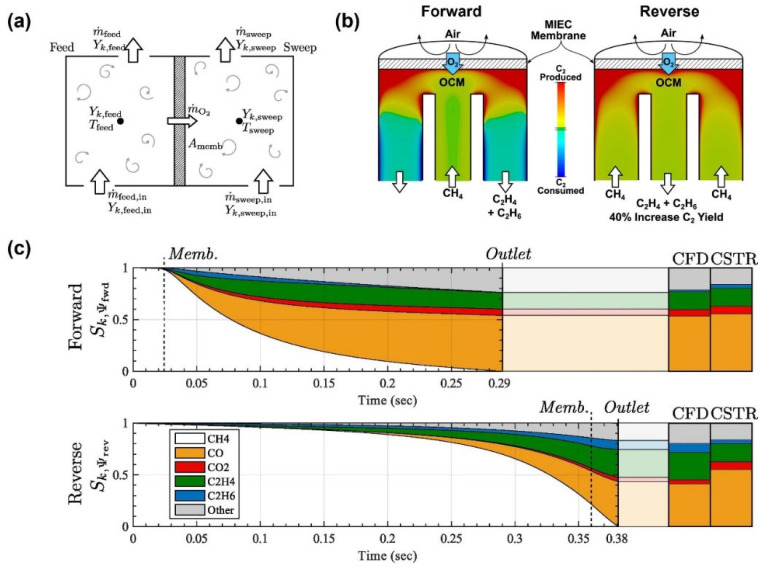
(**a**) Schematic diagram of the coupled two-chamber CSTR model. (**b**) Contours of the production rate of C_2_H_6_ for the forward and reverse button-cell reactor flow configurations. (**c**) Local selectivity of C-products in the forward and reverse button-cell reactor flow configurations. Reprinted with permission from Ref. [107]. Copyright 2019 Elsevier.

In recent years, as CFD technology has become more established in MIEC membrane reactor applications, many researchers have extended its use to study various reactions. Habib et al. [105] successfully applied CFD to predict the combustion characteristics of syngas (a mixture of CO and H_2_) in MIEC membrane reactors. They conducted detailed simulations on the effects of inlet temperature, CO_2_ recirculation, fuel composition, and the sweep gas flow rate. In another study [106], Nemitallah investigated the characteristics of oxygen permeation and the partial oxidation of methane (POM) in a catalytic membrane reactor (CMR), with a focus on optimizing reactor performance to enhance syngas yield. The study revealed that the membrane surface temperature and the oxygen partial pressure gradient drove oxygen permeation. Factors such as the sweep gas flow rate, feed gas flow rate, and flow configuration significantly influenced reaction efficiency and selectivity. The counter-current operation outperformed the co-current configuration, achieving a higher selectivity for CO and H_2_, which reached 89% and 73%, respectively. Sommer et al. [107] studied the performance of MIEC membrane reactors in partial oxidation reactions by establishing a continuously stirred tank reactor (CSTR) and a CFD model (Figure 15a), with a focus on enhancing the selectivity and yield of C_2_ products during the oxidative coupling of methane (OCM). The CSTR model was employed to quickly evaluate optimal operating parameters, while the CFD model incorporated detailed characteristics of gas-phase reaction kinetics and oxygen permeation. The effects of the reaction location of O_2_ and CH_4_, the available reaction time, and the residence time of product formation on reactor performance were investigated by comparing the forward and reverse button-cell reactor flow configurations (Figure 15b). By implementing a reverse flow configuration, the model successfully reduced the reaction time for CH_4_ and O_2_ mixing, thereby inhibiting the further oxidation of C_2_ products and increasing their yield by 40% (Figure 15c). Nonetheless, the CSTR model that they established assumes the complete mixing of gases within the reactor, which may not hold true in practical applications. In actual reactors, there may be instances of non-uniform mixing, which can affect the reaction rate and product distribution.

Hydrogen, as a carbon-free energy carrier, has garnered significant attention, and water splitting via catalytic processes is widely studied for its environmental benefits and potential [108]. Coupling water splitting with MIEC membrane reactors can significantly enhance hydrogen yield through efficient oxygen extraction. Zhao et al. [109] conducted a numerical simulation study using a CFD model based on La_0.7_Sr_0.3_Cu_0.2_Fe_0.8_O_3−δ_ (LSCuF–7328) membrane material, exploring the coupling of water splitting with methane combustion in an OTR. The results demonstrated that methane combustion significantly boosted oxygen permeation and hydrogen production. By reducing the oxygen partial pressure on the sweep side and releasing heat to lower permeation resistance, the hydrogen yield increased by approximately threefold. However, excessively high methane concentrations reduced conversion efficiency, highlighting the oxygen permeation capacity as the critical limiting factor. Optimizing the sweep gas flow rates, methane content, and fuel composition could improve the temperature distribution and reaction efficiency. Bittner et al. [110] compared various reactor designs, including parallel flow, vertical impinging flow, and counterflow designs, revealing the significant impact of geometric factors on membrane performance. For example, the counterflow design demonstrated superior hydrogen production and thermal stability due to a more uniform oxygen transport distribution and reduced temperature gradients.

Ben-Mansour et al. [111] were the first to propose the feasibility of using liquid fuel for oxy-fuel combustion in an OTR. Using a BSCF ionic transport membrane, they demonstrated oxygen-enriched methanol combustion in an OTR and investigated key parameters affecting oxygen permeation and combustion characteristics. A validated numerical model revealed that an optimal combination of the inlet flow rate (0.001 kg s^−1^) and sweep flow rate (7 × 10^−5^ to 1 × 10^−4^ kg s^−1^) could maximize oxygen permeation and combustion efficiency. A moderate increase in the sweep flow rate enhanced the oxygen permeation driving force, although an excessive sweep flow caused flame dilution and temperature reductions. Methanol, as a liquid fuel, exhibited excellent combustion performance in the OTR, making it promising for low-emission combustion applications in gas turbines and boilers. This study provides theoretical support for developing clean and efficient OTR systems, highlighting the potential of membrane technology in carbon reduction and clean energy applications.

CFD studies on MIEC membrane reactions can be combined with other membrane separation processes. Habib et al. [112] extended CFD applications to a mixed-membrane reactor, numerically integrating an MIEC membrane reactor with a polymeric membrane unit to develop a hybrid polymeric–ceramic membrane reactor for enriched oxygen combustion. This reactor used the polymeric membrane for pre-oxygen enrichment, with the oxygen-enriched air from the polymeric membrane fed into the MIEC membrane reactor unit for oxygen separation and pure oxygen combustion. The results showed that, compared to using air, the enriched oxygen feed significantly improved the combustion performance within the reactor while reducing preheating and pumping power demands, lowering energy requirements, and enhancing oxygen combustion efficiency, highlighting the advantages of a hybrid reactor system.

### 4.3. CFD Studies of Membrane Module Design

In the above sections, we delved into the microscale applications of CFD in the study of MIEC oxygen-permeable membranes, including detailed simulations of heat and mass transfer phenomena, as well as its crucial role in the design of membrane reactors. These microscale studies provide a solid theoretical foundation and abundant data support for further understanding the macroscopic behavior of membranes. However, research on MIEC oxygen-permeable membranes is not confined to the microscale. The true value lies in how these microscale mechanisms can be integrated with macroscopic system design in practical engineering applications. Therefore, we expand our perspective to the more complex module and system levels to explore the application of CFD in integrated systems comprising a membrane module, which is essential for improving efficiency and scalability in industrial applications.

**Figure 16:**
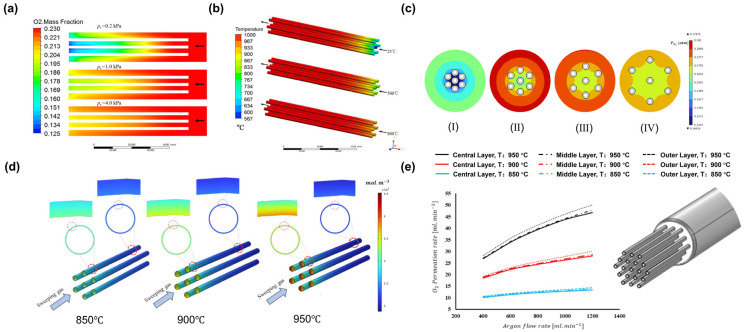
Contours of (**a**) oxygen mass fraction profile and (**b**) temperature profile, reprinted with permission from Ref. [113], copyright 2021 Elsevier. (**c**) Oxygen partial pressure profile, (**d**) oxygen vacancy concentration profile, and (**e**) average oxygen permeation flux of hollow fiber membranes in different layers of the module at different operating temperatures and sweeping gas flow rates, reprinted with permission from Ref. [114], copyright 2024 Elsevier.

However, CFD research on membrane modules has focused mainly on other membrane materials, such as pervaporation membranes, molecular sieve membranes, and other gas separation systems. The application of CFD in MIEC oxygen-permeable membrane modules is relatively rare. Feng et al. [113] utilized CFD to establish a three-dimensional model of an LSCF membrane module assembled with seven hollow fiber membranes. They used user-defined functions (UDFs) to calculate the oxygen separation rate on the membrane. They investigated the impact of operating conditions (temperature, pressure, and vacuum) on the performance of the membrane module. The simulation results indicated that increasing the vacuum on the permeate side was far more effective in enhancing the oxygen separation rate than increasing the pressure on the feed side (Figure 16a). Additionally, preheating the feed gas significantly improved the oxygen separation performance of the module (Figure 16b). Therefore, it is feasible to preheat the feed gas by using a heat exchanger to recover thermal energy from the exhaust gas or oxygen product.

In another study [114], the authors used COMSOL Multiphysics software to develop a multiphysics model that simulates the behavior of the LSCF membrane module under various operating conditions, aiming to optimize the stack structure and improve separation efficiency. The results showed that the temperature and sweep gas flow rate significantly affected oxygen permeation flux, with higher temperatures and flow rates enhancing permeation efficiency. Increasing the distance between hollow fiber membranes effectively increased the oxygen partial pressure; however, there was no noticeable change in the oxygen partial pressure when the distance was overextended (Figure 16c). Additionally, increasing the number of hollow fiber membranes increased the total permeation rate. However, the permeation flux per fiber decreased due to variations in the oxygen partial pressure distribution within the module. To further optimize membrane module performance, the study analyzed the distribution characteristics of the oxygen partial pressure and vacancy concentration within the module (Figure 16d). The findings indicated that the outer fiber layers exhibited a higher oxygen permeation flux (Figure 16e). This suggests that effective control over fiber layout and packing density is essential for enhancing overall module performance.

This research demonstrates that selecting the appropriate hollow fiber layout and operating parameters can achieve optimal permeation efficiency, offering valuable guidance for designing, optimizing, and scaling MIEC oxygen-permeable membrane modules, thereby accelerating the industrial application of oxygen separation. However, the studies conducted by Feng and Xue both have limitations, and it should be noted that this model is only applicable to current small-scale hollow fiber membrane modules. For large reactors, where the flow regime is mostly turbulent, it is necessary to consider radial heat and mass transfer issues when designing large hollow fiber membrane modules. This greatly increases the complexity and computational load of simulations and is currently a significant limitation of CFD in membrane module research.

The pilot-scale amplification of membrane modules is not simply a matter of geometric similarity scaling principles. In laboratory-scale membrane modules, fluid flow may be in a laminar state, where the flow patterns are relatively simple and easy to predict and control. However, during pilot-scale amplification, due to the increase in size and flow velocity, the flow pattern may shift to a turbulent state. The flow characteristics under turbulent conditions are significantly different from those under laminar flow conditions, with changes in the fluid velocity distribution and pressure loss, leading to differences in the mass and heat transfer effects. Additionally, research on the geometric configuration of membrane modules is limited. To further enhance the performance of membrane modules, it is not only necessary to optimize their operating conditions but also to design their geometric configuration reasonably. Specifically, even minor changes in geometric parameters such as the diameter, length, spacing, packing density, arrangement, and overall layout of hollow fiber membranes can lead to significant performance differences.

**Figure 17:**
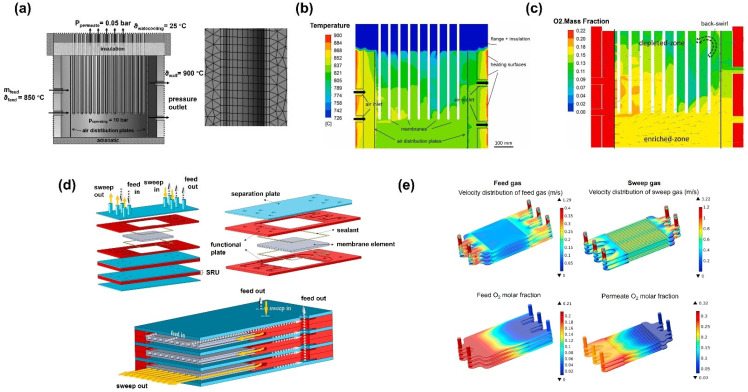
(**a**) Schematic diagram of the tube membrane module. (**b**) Contours of temperature profile. (**c**) Contours of oxygen mass fraction, reprinted with permission from Ref. [115], copyright 2019 Elsevier. (**d**) Schematic diagram of the planar membrane module. (**e**) Contours of velocity profile and oxygen distribution, reprinted with permission from Ref. [116], copyright 2019 Elsevier.

Nauels et al. [115] investigated membrane modules for pilot-scale oxygen production, and they utilized CFD to study the oxygen production process of a membrane reactor consisting of 298 BSCF membrane tubes (Figure 17a). They adopted a three-end operation mode, assuming that the oxygen partial pressure on the permeate side equals the total pressure on the inner side of the tubular membranes. The simulation results were consistent with the oxygen production test results. The CFD simulation revealed the module’s temperature (Figure 17b), flow rate, and oxygen concentration distribution (Figure 17c). The study authors concluded that the decrease in the permeation rate was due to the uneven temperature distribution in the water-cooled section of the module. Therefore, avoiding water-cooled flanges and eliminating temperature gradients along the membrane to prevent membrane tube rupture during experiments are of great importance for membrane module design.

Schulze-Küppersa et al. [116] developed a large-scale planar oxygen transport membrane module for oxy-fuel combustion using LSCF material (Figure 17d), demonstrating scalability and high oxygen permeation efficiency. Through finite element (FEM) modeling and CFD analysis, they optimized the embedded flow channel structure of the membrane. Under a pressure of 5 bar, the module achieved an oxygen concentration of 27% and an oxygen recovery rate of 86% while withstanding high-temperature and high-pressure conditions. The symmetrical structural design ensured both chemical stability and high permeation performance. Mechanical stability was enhanced by combining a thin membrane layer (20 µm thick) with a support layer (38% porosity). The manufacturing process employed a blade-casting technique to progressively fabricate the membrane and support layers, ensuring uniformity and minimizing defects. Through a combination of high-temperature sintering and integrated processes, the scalable production of membrane modules was successfully achieved, demonstrating feasibility and consistency for large-scale manufacturing. Additionally, the membrane modules maintained stability under high-pressure conditions, with a low risk of rupture. This study provides robust technical support for the industrial implementation of oxy-fuel combustion and carbon capture technologies.

## 5. Challenges and Prospects of CFD in MIEC Oxygen-Permeable Membranes

### 5.1. Challenges

The application of CFD in research on MIEC oxygen-permeable membranes has made significant progress in understanding transport mechanisms, reactor design, and performance optimization. However, the application of CFD still faces numerous challenges that limit its widespread use in large-scale industrial applications.

The core of MIEC oxygen-permeable membranes lies in their complex multiphysics interactions. Oxygen permeation is constrained by the physicochemical properties of the membrane material, including the oxygen vacancy concentration, electronic conductivity, and surface exchange reaction rates. However, current CFD models often simplify real-world conditions, for example, by assuming constant surface reaction rates or neglecting certain nonlinear effects. These approximations can lead to deviations under specific conditions. Moreover, developing fully coupled multiphysics models is highly challenging, requiring greater mathematical precision and computational power to solve complex equation systems, which places a high demand on researchers.

Parameter acquisition and validation are other key challenges in CFD applications. Accurate simulations depend on extensive input parameters, such as material properties, reaction rate constants, and diffusion coefficients. However, these parameters are often difficult to measure accurately in high-temperature and highly oxidative environments, especially for newly developed MIEC membrane materials. The uncertainty in these parameters can significantly affect the accuracy of model predictions. Furthermore, CFD model validation requires a comparison with experimental data. However, obtaining experimental conditions that align perfectly with simulation conditions remains a significant challenge; thus, the gap between experimental and simulated data limits further model optimization.

In industrial applications, the computational cost and scalability of CFD simulations are particularly prominent. To capture details such as local oxygen concentration polarization, temperature fluctuations, and pressure distributions, CFD models require high-resolution grid division and small time steps, significantly increasing computational complexity. Especially when simulating large membrane modules, the computational load for handling multi-module flow fields and complex reactor internal dynamics is considerable. Researchers often face trade-offs between model detail and computation time, restricting CFD’s applicability under industrial-scale conditions. Moreover, during the scale-up process from laboratory to industrial systems, changes in flow patterns, temperature distribution, and membrane performance may occur, and CFD models often show limitations in addressing this complexity.

### 5.2. Prospects

Despite these challenges, the future prospects of CFD in MIEC oxygen-permeable membrane research remain promising. Emerging computational technologies, particularly machine learning (ML) [117,118,119,120] and digital twin (DT) techniques [121], are poised to overcome these barriers by enabling intelligent acceleration, physics-aware automation, and cyber–physical synergy in membrane research. At the same time, the combination of molecular dynamics (MD) [122,123] can significantly enhance the understanding of the correlation between the microscopic mechanism and macroscopic properties of membrane materials. These tools not only enhance the predictive power of CFD but also unlock unprecedented capabilities in adaptive system control and inverse design. Although there has been minimal use of these computational tools to assist CFD in MIEC membrane research thus far, we can draw on studies from other membrane fields to explore how these disruptive innovations can propel MIEC membrane engineering into a new era of efficiency and innovation.

#### 5.2.1. ML in CFD-Based Membrane Design

The integration of ML with CFD has ushered in a new paradigm for optimizing MIEC oxygen-permeable membranes, as detailed below.

On the one hand, ML can efficiently analyze and mine large volumes of experimental data and CFD simulation results [124]. In MIEC oxygen-permeable membrane research, a vast amount of data are generated, covering flow, temperature, and concentration fields under diverse operating conditions, as well as membrane performance parameters. By utilizing ML algorithms, such as neural networks and decision trees, potential patterns and correlations in the data can be rapidly identified. For instance, when the microstructural features and macroscopic performance data of membranes are fed into an ML model, the model can learn the mapping between them. This provides vital guidance for optimizing membrane microstructures to enhance performance, bypassing the traditional, time-consuming trial-and-error approach [125].

On the other hand, ML can be combined with CFD models to enhance and optimize them [126]. When simulating the complex multiphysics processes of MIEC oxygen-permeable membranes, CFD models often involve assumptions and simplifications, leading to discrepancies between simulation results and actual conditions. ML can calibrate and optimize uncertain parameters in CFD models through Bayesian methods, improving predictive accuracy. Moreover, ML can construct surrogate models to accelerate CFD simulations. By training surrogate models (e.g., Fourier neural operators (FNOs) and convolutional neural networks (CNNs)) based on high-fidelity CFD data, ML can significantly reduce computational costs while maintaining acceptable accuracy. This enables the rapid screening and evaluation of different design parameter combinations [127], thereby boosting the efficiency of membrane design based on CFD. These models could also be applied in real-time performance monitoring and control for industrial applications.

Although ML is still in the exploratory stage in this field, some studies have begun applying it to MIEC oxygen-permeable membrane design. For example, Schlenz et al. [128] used ML to predict the chemical composition of cobalt-free and rare-earth-free perovskite oxygen separation membranes and to optimize their electronic and ionic conductivities. As ML algorithms continue to improve and high-performance computing resources become more abundant, ML is expected to play a more pivotal role in MIEC oxygen-permeable membrane design based on CFD, strongly supporting the development of high-performance and efficient oxygen-permeable membranes.

#### 5.2.2. DTs in CFD-Based Membrane Design

DT technology, by creating a virtual copy of a physical system, has become a new way to change the engineering design and operation of complex systems, providing a closed-loop support for the smart design and operation of MIEC membranes.

By combining sensor data with CFD, DTs can monitor membrane performance in real time and enable predictive maintenance [129]. These data can be fed into CFD to update and optimize the virtual model. This closed-loop feedback mechanism ensures the accuracy of CFD predictions and their relevance to actual membrane operation. During the oxygen separation process using MIEC membranes, DTs can monitor parameters such as temperature, pressure, and oxygen concentrations in real time. If deviations from the CFD-based performance expectations are detected, the model can be adjusted to account for any changes in the membrane’s physical or chemical properties, thereby optimizing operating conditions in a timely manner. At the same time, the closed-loop nature of DTs (monitoring–analysis–decision making) can achieve adaptive control, and hybrid models based on CFD-ML can adjust the flow field distribution in the reactor in real time, avoiding local concentration polarization. Such systems have been verified in the aerospace and energy fields [130,131].

In addition, engineers can predict potential failures and degradation mechanisms by using digital twins to simulate the long-term behavior of MIEC membranes based on CFD analysis and real-time data. This enables the implementation of predictive maintenance strategies, significantly extending the membrane’s lifespan and reducing operating costs.

#### 5.2.3. MD in CFD-Based Membrane Design

In addition to using ML and DT to assist the CFD process, methods such as MD or Monte Carlo simulations can be used to study the migration mechanism of ions in MIEC materials. Multiscale modeling integrating MD and CFD can more accurately describe the migration path of oxygen vacancies on the membrane surface and in the bulk. For example, drawing on the multiphysics coupling method in flame spray pyrolysis reactors (such as the simulation of turbulence–chemical reaction–particle nucleation) [132], a dynamic coupling model of oxygen vacancy diffusion and surface exchange reactions in MIEC membranes can be constructed.

MD provides microscopic information, such as the position, velocity, force, and energy of molecules or atoms. Macroscopic parameters (density, viscosity, diffusion coefficients, thermal conductivity, etc.) obtained through statistical analysis can serve as input for CFD. Based on the microscopic regions and physical processes of MD, the channel geometry, inlet and outlet positions, and velocity information can be converted into boundary conditions for CFD. Local flow field information from CFD can also serve as external environmental conditions for MD.

## 6. Conclusions

In summary, in this review, we comprehensively examine the applications and advancements of CFD in research on MIEC oxygen-permeable membranes. By accurately simulating heat and mass transfer processes, as well as the design of reactors and membrane modules, CFD has become an indispensable tool for improving the performance of MIEC membranes. Its powerful capabilities enable researchers to investigate complex physical and chemical interactions, uncovering key factors that influence membrane performance, such as temperature distributions, oxygen partial pressure gradients, and material properties. These insights provide valuable theoretical guidance for optimizing materials, structural designs, and engineering applications.

However, challenges remain in the broader application of CFD. These include the development of sophisticated multiphysics models, uncertainties in obtaining key parameters in high-temperature oxidative environments, and the high computational costs associated with large-scale simulations. These limitations hinder the full realization of CFD’s potential in industrial applications.

Future research should focus on the great potential of emerging computational technologies, such as ML, DTs, and MD simulations, in enhancing the predictive capabilities of CFD for MIEC oxygen-permeable membrane research, as well as in adaptive control and inverse design. ML can optimize CFD simulations, DTs can assist in real-time monitoring and predictive maintenance, and a combination of MD and CFD can provide a deeper understanding of the correlation between the microscopic mechanisms and macroscopic properties of membrane materials, thus promoting the development of MIEC membrane technology.

## Figures and Tables

**Table 1:** The mathematical models used in the CFD simulation of MIEC membranes.

Mathematical Model	Model Equation	Application Scenarios
Wagner theory model [43] (2014)	$J_{O_{2}}=\frac{1}{4^{2}F^{2}L}\int_{\mu_{O_{2}\left(Ⅱ\right)}}^{\mu_{O_{2}\left(Ⅰ\right)}}t_{ion}t_{e}\sigma^{tot}d\mu_{O_{2}}$	This model is suitable for thick membranes, single-phase MIEC membranes, and steady-state high-temperature operations.
Xu–Thomson model [44] (1999)	$J_{O_{2}}=\frac{k_{r}/k_{f}\left({P_{O_{2}}^{\prime \prime }}^{-1/2}-{P_{O_{2}}^{\prime }}^{-1/2}\right)}{\left(1/k_{f}{P_{O_{2}}^{\prime }}^{1/2}\right)+\left(2L/D_{v}\right)+\left(1/k_{f}{P_{O_{2}}^{\prime \prime }}^{1/2}\right)}$	This model is suitable for studies where variations in the oxygen vacancy concentration depend on the oxygen partial pressure.
Kim model [46] (1999)	$\frac{{4nLJ}_{O_{2}}}{\langle c_{ion}D_{a}\rangle }=\ln\frac{\sqrt{P_{O_{2}}^{\prime }/P_{O_{2}}^{o}}-2J_{O_{2}}/c_{ion}^{\prime }k}{\sqrt{P_{O_{2}}^{\prime \prime }/P_{O_{2}}^{o}}-2J_{O_{2}}/c_{ion}^{\prime \prime }k}$	This model is suitable for disc membranes, assuming that the oxygen vacancy density at the membrane interface does not significantly vary with the oxygen partial pressure.
Kim model [46] (1999)	$\frac{2nN_{O_{2}}}{\pi \langle c_{i}D_{a}\rangle w}\ln\frac{r_{1}}{r_{2}}=\ln\frac{{\left(P_{O_{2}}^{\prime }/P_{O_{2}}^{o}\right)}^{n}-N_{O_{2}}/\pi r_{1}wc_{i1}k}{{\left(P_{O_{2}}^{\prime \prime }/P_{O_{2}}^{o}\right)}^{n}+N_{O_{2}}/\pi r_{2}wc_{i2}k}$	This model is suitable for tubular membranes, assuming that the oxygen vacancy density at the membrane interface does not significantly vary with the oxygen partial pressure.
Tan and Li model [47] (2002)	$J_{O_{2}}=\frac{k_{r}\left({P_{O_{2}}^{\prime }}^{1/2}-{P_{O_{2}}^{\prime \prime }}^{1/2}\right)}{\frac{2k_{f}\left(R_{o}-R_{\mathrm{in}}\right){P_{O_{2}}^{\prime }}^{1/2}{P_{O_{2}}^{\prime \prime }}^{1/2}}{D_{v}}+\frac{R_{m}{P_{O_{2}}^{\prime }}^{1/2}}{R_{in}}+\frac{R_{m}{P_{O_{2}}^{\prime \prime }}^{1/2}}{R_{o}}}$	This model is suitable for hollow fiber membranes.
Zhu model [48] (2012)	$J_{O_{2}}=\frac{∆\mu_{O_{2}}^{tot}}{4^{2}F^{2}}\frac{1}{r_{tot}}$	This model is suitable for single- and dual-phase disc membranes, where both surface exchange reactions and bulk diffusion limit the process.
Liu model [50] (2005)	$J_{O_{2}}=Ak\left(T\right)G\left[{\left(P_{O_{2}}^{\prime }\right)}^{n}-{\left(P_{O_{2}}^{\prime \prime }\right)}^{n}\right]$ (*n* < 1) $J_{O_{2}}=Ak\left(T\right){\left[\frac{P_{O_{2}}^{\prime }}{P_{O_{2}}^{\prime \prime }}\right]}^{n}$ (*n* > 0)	This model is suitable for making quick predictions of oxygen permeation performance, particularly for preliminary studies in large-scale industrial applications.

## Data Availability

No new data were created or analyzed in this study. Data sharing is not applicable to this article.
